# Quantum-Mechanical Calculations on Molecular Substructures Involved in Nanosystems

**DOI:** 10.3390/molecules191015468

**Published:** 2014-09-26

**Authors:** Beata Szefler, Mircea V. Diudea

**Affiliations:** 1 Department of Physical Chemistry, Collegium Medicum, Nicolaus Copernicus University, Kurpińskiego 5, Bydgoszcz 85-096, Poland; 2 Faculty of Chemistry and Chemical Engineering, Babes-Bolyai University, Arany Janos street 11, Cluj-Napoca RO-400028, Romania

**Keywords:** *ab initio*, HOMA, NICS, nanotube junction, circulene, fullerene, polybenzene, periodic network, vibrational spectra

## Abstract

In this review article, four ideas are discussed: (a) aromaticity of fullerenes patched with flowers of 6-and 8-membered rings, optimized at the HF and DFT levels of theory, in terms of HOMA and NICS criteria; (b) polybenzene networks, from construction to energetic and vibrational spectra computations; (c) quantum-mechanical calculations on the repeat units of various P-type crystal networks and (d) construction and stability evaluation, at DFTB level of theory, of some exotic allotropes of diamond D_5_, involved in hyper-graphenes. The overall conclusion was that several of the yet hypothetical molecular nanostructures herein described are serious candidates to the status of real molecules.

## 1. Introduction

Among the carbon allotropes discovered in the nano-era, fullerenes (zero-dimensional), nanotubes (one dimensional), graphene (two dimensional), spongy carbon and hyper-diamonds (three dimensional) are the most challenging [1,2,3]. Inorganic compounds including oxides, sulfides, selenides, borates, silicates, *etc*., of many metals, have also found applications as nano-structured functional materials [4,5]. It is nowadays a common fact that nanotechnologies and nanomaterials have a great impact in the development of almost every industry [6,7,8,9,10]. The demands for new materials with adjustable properties have increased the interest for the study of possible nanomaterial precursors.

The polyaromatic hydrocarbons, PAHs, gained a new dimension by their recognition as precursors of graphene, and hence, of any of the abovementioned carbon allotropes. The progress in electronics and microscopy made possible new investigations and applications of very promising molecular structures. Quantum calculations would be useful in providing the theoretical background for new syntheses and applications.

This review article is organized as follows: after a very short introduction, the second section introduces to the design of new fullerenes patched with various circulenes, called here flowers, and their aromaticity *vs*. stability/reactivity is discussed in terms of HOMA and NICS parameters, based on Hartree-Foch and DFT calculations. Section 3 deals with polybenzenes, periodic nanostructures together with their repeating units, as finite molecules, optimized also at the Hartree-Foch and DFT levels of theory. In Section 4, some 3-periodic nanostructures built up by small units designed by opening spherical fullerenes are presented. In Section 5, some exotic molecules, involved in 2D- and 3D-nanosystems, are presented and their relative stability is evaluated at the DFTB level of theory. The review is ends with conclusions and references.

## 2. Circulene Patched Fullerenes

The specific properties of fullerene-based nanomaterials are mainly due to their π electron systems, so investigating the aromatic character of fullerenes and their precursors may offer a good insight into the nanomaterial properties. The aromatic character is a molecular property, conditioned by energy, electronic structure, magnetic response, geometric characteristics or chemical behavior [11,12,13]. Accordingly, various orderings are expected in sets of molecules with respect to different parameters, broadly called aromaticity criteria.

In the *energetic criterion*, a more aromatic character means a more stable structure [13]. Despite the fact resonance energy [14,15,16] plays an important role in stabilizing (at least) planar polyhex structures, the strain appearing in fullerenes, nanotubes, *etc.*, will decide the overall stability (and reactivity) of such molecules.

The *electronic criterion* requires π-electron delocalization [11,12] (and bond length equalization). However, aromaticity is a local property, in the sense that small benzenic or naphthalenic units, rather than larger circuits, will manifest in chemical reactions. The π-electron distribution can be presented in terms of the *numerical Kekulé* *valence* *structures* [17,18,19,20,21,22,23,24] that, in contrast to *geometrical* Kekulé structures (e.g., the icosahedral C_60_ has 12,500 such structures), enable the construction of a single numerical structure to account for the superposition of the geometrical Kekulé structures, as in the Clar representation [25,26]. With regard to stability, a higher Kekulé structure count *K* is associated with a higher stability [11,27]. There are, however, twenty C_60_ isomers with *K* > 12,500, although they are less stable [28,29,30]. As mentioned above, the strain in the σ-frame could be a more important energetic factor, particularly in non-planar molecules like fullerenes, nanotubes, *etc.*, where it may change the expected (by aromaticity) ordering. Thus, *K* alone seems not to be a reliable predictor of energetically favorable structures, and the conjugated circuits count has proven to be a more adequate description [11,31,32,33].

The *magnetic criterion* describes the π-electron delocalization, with direct consequences on the magnetic properties, e.g., the diamagnetic susceptibility and NMR chemical shifts. These effects can be rationalized in terms of ring currents induced by the external field. Ring-current effects have long been recognized as important indicators of aromaticity. Depending on the number of π*-*electrons, diatropic or paratropic ring currents may occur. In fullerenes, enhanced aromaticity, as assessed by magnetic criteria, does not necessarily imply additional stabilization. The considerable strain of the σ-frame may dominate the stability and reactivity [11].

The *structural/geometric criterion* predicts for C_60_ a pronounced bond-length alternation between [6,6]- and [5,6]-bonds [11]. Experimental data have also shown that, in neutral fullerenes, the [6,6]-bonds (i.e., the bonds shared by two hexagons) are shorter than [5,6]-bonds [34,35,36]. The bond-length alternation is strongly supported by the regioselectivity of addition reactions [13]. Based on the geometric criterion, Krygowski has proposed an index of aromaticity, called harmonic oscillator model of aromaticity (HOMA) [37,38,39,40,41]. It is calculated on the difference between the actual CC and the CC equalized bond lengths.

To conclude, aromaticity is a multi-dimensional phenomenon [42,43,44]. Fullerenes rather show an alkenic character [28], with additions being the most favored reactions. The electron deficiency of fullerenes results from the presence of the 12 pentagons (appearing as defects in the graphite sheet) needed to close the cage.

**Figure 1:**
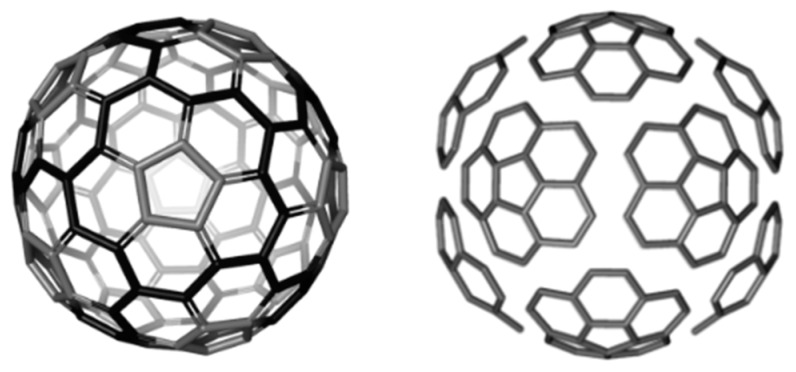
The [5:6_5_] patch in fullerenes: joint patch in C_140_ (**left**); disjoint patch in C_240_ (**right**).

A circulene is a flower-like molecule, with a core and surrounding petals and general formula [*n*:(*p*_1_,*p*_2_)*_n_*_/2_], where *n* is the size of the core polygon and *p_i_* are the polygonal petals. For *n* < 6, the molecule has a bowl-shaped geometry whereas for *n* > 6 it is saddle-shaped [1,2,45]. The bowl-shaped circulenes are potentially useful in the direct synthesis of fullerenes [46,47] while the saddle-shaped ones would appear as patches in the foamy structures of spongy carbon [48,49]. The idea of increasing the stability of fullerenes tessellated by disjoint circulenes/flowers originates in the classical texts of Clar [25,26] who postulated *disjoint benzenoid rings* as a criterion for aromatic conjugation [50]. Figure 1 shows two types of fullerene covering, one with joined patches and the other one with disjoined patches.

The aromatic character of various flowers, with the core being either a hexagon or an octagon, has been evaluated by means of geometric (HOMA index), energetic (heats of formation) and magnetic (NICS index [51] and exaltation of magnetic susceptibility) criteria.

### 2.1. Circulenes with Hexagonal Core

**Figure 2:**
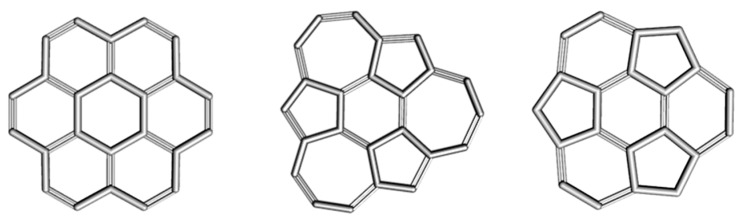
Circulenes with hexagonal core: coronene [6:6_6_]_24 (**left**); isocoronene [6:(5,7)_3_]_24 (**middle**) and sumanene [6:(5,6)_3_]_21 (**right**).

The circulenes herein considered [52]: coronene [6:6_6_], isocoronene [6:(5,7)_3_] and sumanene [6:(5,6)_3_] are shown in Figure 2.

#### 2.1.1. Global Stability

In order to evaluate the stability of the considered polycyclic compounds, the HOMO-LUMO HL gap and total energy per C-atom were computed [52] (Table 1). HOMO-LUMO gap may be considered as an approximation to the chemical hardness and also an indicator of the molecular kinetic stability.

**Table 1:** Total energy, E_tot_ (au), total energy per C-atom, E_tot_/C (au), and HOMO-LUMO HL gap (eV), computed at HF/6-31G(d) level of theory [52].

Structure	Symmetry	E_tot_	Etot/C	HL Gap
Coronene [6:6_6_]	D_6h_	−915.953	−38.164	8.960
Isocoronene [6:(5,7)_3_]	C_s_	−915.780	−38.156	7.260
Sumanene [6:(5,6)_3_]	C_3v_	−802.190	−38.200	10.160

Larger values of HOMO-LUMO gap are found for coronene and sumanene that suggest a higher stability for these two experimentally known molecules.

#### 2.1.2. Aromaticity

The local aromaticity of these circulenes can be evaluated by calculating the NICS(0) and NICS(1) indices for every ring of the polycyclic hydrocarbons [52]. Results of these calculations are shown in Table 2, Table 3, Table 4, Table 5 and Table 6.

For coronene, the NICS data show a pronounced aromatic character of the outer benzenic rings and lower aromatic or even non-aromatic character of the core hexagon. These data support the “radialene”-structure of coronene, as depicted in Figure 2 (left). The HOMA values also show an enhanced aromaticity on the outer rings [52].

It should be noted that coronene itself is not a totally resonant hydrocarbon [11,53] because every Kekulé structure leaves some carbon atoms outside the sextet rings. However, Clar [26] proposed that if the three sextets of coronene can migrate into the neighboring rings, an extra ring current would emerge. The sextet migration current can be taken as an argument in favor of the enhanced aromaticity of coronene (compared to some other polycyclic hydrocarbons, e.g., naphthalene and anthracene) [11].

**Table 2:** The NICS(0), NICS(1) and HOMA values calculated for the B3LYP/6-31G(d) optimized geometry of coronene [52].

Coronene [6:6_6_]	NICS(0)	NICS(1)	HOMA
R[6] core	−0.009	−4.429	0.618
R[6] petal	−10.406	−12.453	0.764

**Table 3:** Values of NICS(0), NICS(1) and HOMA calculated for the B3LYP/6-31G(d) optimized geometry of isocoronene [52].

Isocoronene [6:(5,7)_3_]	NICS(0)	NICS(1)	HOMA
R[6] core	−2.908	−5.156	0.867
R[7] petal	0.394	−2.712	0.013
R[5] petal	−3.136	−5.637	−0.036

**Table 4:** The NICS(0), NICS(1) and HOMA values calculated for B3LYP/6-31G(d) optimized geometry of sumanene [52].

Sumanene [6:(5,6)_3_]	NICS(0)	NICS(1)	HOMA
R[6] core	−2.767	−10.385	0.708
R[6] petal	−10.080	−16.892	0.925
R[5] petal	3.189	−5.192	−1.955

**Table 5:** Total energy, E_tot_ (au), total energy per C-atom, E_tot_/C (au), and HL gap (eV), of the tetrahedrally spanned fullerenes (as hydrogen-ended structures), patched by coronene Cor and sumanene Sum; reference: C_60_(*I_h_*) fullerene [52].

Structure	Theory Level	E_tot_	E_tot_/C	HL Gap
Cor_T-84	HF/6-31G(d,p)	−3194.384	−33.275	7.347
Sum_T_84		−3155.466	−38.028	7.562
C_60_(*I_h_*)		−2271.830	−37.864	7.418
Cor_T-84	B3LYP/6-31G(d,p)	−3215.331	−35.333	2.268
Sum_T_84		−3214.968	−38.273	2.520
C_60_(*I_h_*)	B3LYP/6311 + G(d,p)	−2286.610	−38.110	2.724

Computations of the NICS(0) index for [6:(5,7)3] isocoronene (Figure 2, middle) provide close values for the central 6-membered and the 5-membered rings of this polycyclic structure, the rather low negative values indicating a low aromatic character [52] (Table 3). The NICS(0) positive values of the 7-membered rings suggest a non-aromatic character. The NICS(1) index is often employed as an indicator of the π-electron delocalization; in the case of 6- and 5-membered rings of isocoronene, it provides “more negative” values. The enhanced values are attributed by Fowler *et al*. [54] to the electron flow through the outside perimeter of the rings. On the other hand, the HOMA values show a different trend compared to both of the NICS indices, suggesting a more pronounced aromatic character of the central benzenic ring (see also [55]).

The values of the NICS(0) and NICS(1) indices for sumanene (Figure 2, right) correspond to an anti-aromatic character of the pentagons, a strong aromatic character of the outer benzene rings and a lower aromatic character of the core R[6] ring (Table 4). The HOMA data closely parallel their NICS counterparts [52].

**Table 6:** Aromaticity (HOMA and NICS indices) and strain (POAV, kcal/mol) of coronene and sumanene patches in the tetrahedrally spanned fullerenes (Figure 3) (optimised at HF/6-31G(d,p); B3LYP/6-31(d,p) levels of theory) [52].

Structure	Substructure	HOMA	Strain	NICS(−1)	NICS(0)	NICS(+1)
Cor_T_84						
HF	R_6,core_	0.525	0.387	−5.258	−0.789	−5.257
	R_6,plane_	0.908	0.709	−16.429	−11.928	−11.342
	R_6,bound_	0.047	2.699	−13.533	−4.518	−1.812
	patch	0.374	2.053			
	molecule	0.348	1.477			
B3LYP	R_6,core_	0.529	0.474	−9.184	−1.749	−2.808
	R_6,plane_	0.804	0.765	−16.555	−12.123	−11.443
	R_6,bound_	0.162	2.543	−14.554	−5.532	−2.259
	patch	0.422	1.938			
	molecule	0.392	1.435			
Sum_T_84						
HF	R_6,Core_	0.849	2.488	−11.775	−2.313	−2.078
	R_6_	0.896	1.169	−16.264	−11.349	−9.801
	R_5_	−1.685	2.357	−7.314	1.773	2.203
	patch	−0.548	1.684			
	molecule	−0.476	1.685			
B3LYP	R_6Core_	0.889	3.158	−11.807	−2.437	−1.798
	R_6_	0.850	1.408	−16.177	−11.067	−9.047
	R_5_	−1.162	2.568	−7.006	2.779	2.117
	patch	−0.296	1.832			
	molecule	−0.229	1.833			

**Figure 3:**
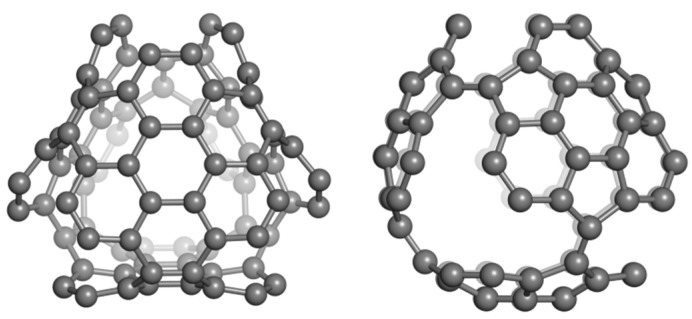
The tetrahedrally spanned fullerenes patched by coronene (Cor_T_84, **left**) and sumanene (Sum_T_84, **right**).

The coronene and sumanene fragments can be inserted into 3D-structures such as the tetrahedrally spanned fullerenes depicted in Figure 3. These structures can be derived from the fullerene C_84_ and were named Cor_T_84 and Sum_T_84, respectively; they can also be considered as junctions of nanotubes [1]. Even there are many tessellation for the tetrahedral nanotube junctions, our option was for these two patches, as they also represent real molecules. Data for these structures (as hydrogen-ended ones) are compiled in Table 5, in comparison to those for all-carbon C_60_(*I_h_*), the most used reference structure in nanoscience [56]. It seems the two tetrahedral structures show a pertinent stability, when compared to that of the reference fullerene, with Sum_T_84 being particularly stable [52]. However, there is no direct comparison with C_60_ since there are known the differences, at least in HL gap, between the all-carbon- and hydrogen-ended structures.

The NICS(0) values in Cor_T_84 (Figure 3, left and Table 6) are in good agreement with those in the free coronene molecule (Table 2). The DFT data show (in general) the same trend, with an even increased negative values of NICS indices. The index NICS(−1) refers to the “inside” while NICS(+1) refers to the “outside” of spanned tetrahedral fullerenes. The NICS(−1) negative values are larger than those provided by NICS(+1), indicating a higher conjugation of π-electrons inside the structure.

The HOMA values calculated for Cor_T_84 exhibit the same trend as the NICS(−1) values, namely the highest aromaticity of the free hexagons R_6,plane_, followed by the R_6,Core_ and finally the bound hexagons. The HOMA index allows calculation for both a patch and the whole molecule (Table 6). However, this geometric criterion must be completed with other criteria of aromaticity when an ordering of molecules is attempted [52].

In Sum_T_84 (Figure 3, right), all the NICS values exhibit the highest aromaticity of the outer R_6_ rings in comparison to the core hexagon (see also Table 4). The pentagons appear rather anti-aromatic by NICS(−1) values but still aromatic by NICS(+1) values, (with lower values in comparison to the core hexagon). In case of HF-data, the HOMA vaues follow the trend of NICS(0) and NICS(−1) values while in the DFT-optimized structure the trend of HOMA values is different from that of NICS data.

The extent of strain, evaluated by POAV1 theory [57], varies among the rings. It is the largest for the bound-hexagons in Cor_T_84 and for the core hexagon and pentagons in Sum_T_84, but these values are even lower than those for C_60_(*I_h_*) (8.256 kcal/mol) because these structures are “opened fullerenes”. The extent of strain for the patch and the whole molecule are irrelevant. Since the NICS and HOMA calculations indicated the presence of some anti-aromatic substructures, it was necessary to recalculate the basic 6-flowers: coronene, isocoronene and sumanene, both in singlet and multiplet states [52] (Table 7).

**Table 7:** Total energy, E_tot_ (in au), total energy per C-atom, E_tot_/C, and HL gap (in eV), (HF and DFT (B3LYP/6-31(d,p)) for the 6-flowers, in singlet and multiplet states (in Italics) [52].

Structure	E_tot_	E_tot_/C-atom	HL gap	E_tot_	E_tot_/C-atom	HL Gap
Theory level	HF			DFT		
[6:6_6_] coronene	−915.640	−38.151	8.956	−922.071	−38.420	4.026
coronene_3	−915.531	−38.147	*6.401* *6.619*	−921.966	−38.415	*1.156* *1.144*
[6:(5,7)_3_] isocoronene	−915.427	−38.143	7.032	−921.909	−38.413	1.933
isocoronene_3	−915.416	−38.142	*6.598* *7.151*	−921.878	−25.608	*1.073* *1.061*
[6:(5,6)_3_] sumanene_2	−800.033	−38.097	*8.378* *8.347*	−805.640	−38.364	*1.619* *2.838*
[6:(5,6)_3_] sumanene_4				−805.588	−38.361	*3.520* *1.408*

The calculations have shown that no important variation in HL gap values appear between the alpha and beta orbitals of the triplet states (in italics) of coronene and isocoronene molecules, as the conjugacy of the pi-electron was not deeply affected.

The sumanene triradical should be non-planar. Planarization induces in-plane symmetry breaking; as a consequence, the sumanene gap value presented in Table 1 was overestimated. The differences in HOMO-LUMO gap of the alpha and beta orbitals, in the higher multiplicity state, clearly indicates a lower conjugasy (and a lower aromaticity) for the sumanene structure.

### 2.2. Circulenes with Octagonal Core

**Figure 4:**
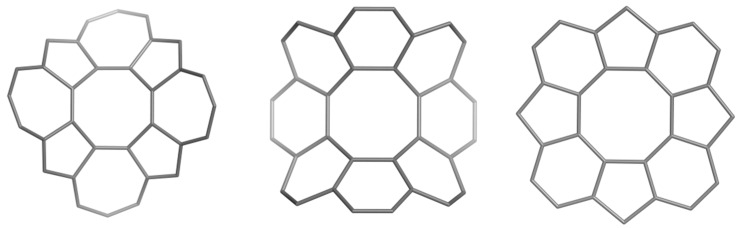
Circulenes with octagonal core: [8:(5,7)_4_]_32 (**left**); [8:6_8_]_32 (**middle**) and [8:(5,6)_4_]_28 (**right**).

In this section, the aromatic character of circulenes with octagonal core and petals consisting of 5-, 7-rings is discussed [58]. The three 8-flowers herein discussed are shown in Figure 4.

The values of HOMA and NICS(0) indices and the magnetic susceptibilities have been computed; in addition, two different isodesmic reactions (for each molecule in Figure 4) have been proposed for calculating the enthalpies of formation. Electronegativity [59], total hardness [59], the electrophilicity index [59] and Fukui functions [60] (for an electrophilic attack) have been computed based on DFT methods.

#### 2.2.1. Enthalpy of Formation (Energetic Criterion)

**Figure 5:**
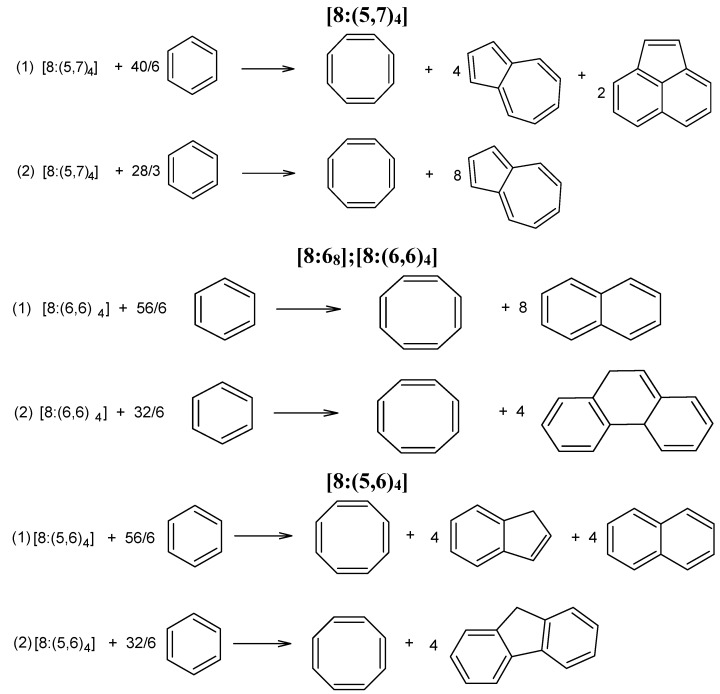
Isodesmic reactions for the enthalpy calculation in 8-flowers [8:(5,7)_4_], [8:6_8_] and [8:(5,6)_4_].

The stability of a polycyclic hydrocarbon can be investigated on the basis of some computed thermodynamic values, particularly the enthalpy of formation. Comparison between the calculated heats of formation of the circulenes [8:(5,7)_4_], [8:6_8_] and [8:(5,6)_4_] and the experimental available data, e.g., for coronene, may lead to valuable conclusions regarding the stability of the three mentioned flowers. In this regard, two isodesmic reaction schemes for each of these circulenes were proposed [58] (Figure 5); the average heat of formation was calculated, as shown in Table 8.

If we compare the results with the experimental heat of formation of coronene (36.4 kcal/mol) [61,62], a significant difference of stability appears (*i.e*., the 8-circulene is far more unstable). The most stable compound, according to these calculations, should be the circulene [8:(5,6)_4_], the one showing a planar structure [58]. The same conclusion was drawn from the single point calculations on the optimized structures at HF/6-311G(d,p)) level of theory.

**Table 8:** Enthalpy of formation, ΔH_f_ (kcal/mol), of 8-flowers (Figure 4) (optimized at the HF/6-311G(d,p) level of theory) [58].

Structure	Isodesmic Reaction	ΔH_f_
[8:(5,7)_4_]	1	390.5
	2	344.9
[8:6_8_]	1	334.9
	2	343.4
[8:(5,6)_4_]	1	219.0
	2	213.2

#### 2.2.2. 8-Flowers as Real Molecules

Tetraoxa[8]circulenes (see [8:(5,6)_4_] flower, Figure 4) attracted a considerable interest of researchers, as they represent promises for blue fluorescent organic light-emitting diodes [63,64,65,66,67,68,69,70,71]. The quantum calculations and vibrational data of these molecules have been reported only recently [63,65,72,73,74]. The synthesis of π-extended tetraoxa[8]circulenes by statistical condensation of 2,3-dialkyl-1,4-benzoquinone with naphthoquinone has been described [63]; their FTIR and Raman spectra have been measured and the assignment of the observed bands in FTIR and Raman spectra was computed at DFT level [72,74]. Note that the simple [8]-circulene, *i.e.*, the pure hydrocarbon [8:(5,6)_4_] was studied earlier by quantum-chemical π-approximation [75] and ab initio methods [76].

#### 2.2.3. NICS(0) Index and Exaltation of Magnetic Susceptibility (Magnetic Criterion)

NICS(0) index (or variants like NICS(0)_πzz_) [77] is widely used as a local descriptor of aromaticity. NICS(0) values were calculated for the 8-membered central ring of the studied circulenes, as well as for the petal-rings (Table 9) [58].

**Table 9:** NICS(0) values for the 8-flowers in Figure 4 (optimized at the B3LYP/6-31G(d) level) [58].

Structure	Core	Petals (5 Atoms)	Petals (6 Atoms)	Petals (7 Atoms)
[8:(5,7)_4_]	−2.330	−4.093 (−3.136)	-	1.842 (0.393)
[8:6_8_]	9.465	-	−7.788/−3.863	-
			(−10.406)	
[8:(5,6)_4_]	8.172	0.686 (3.189)	−6.314 (−10.080)	-

For comparisons, data for the petal rings in circulenes with hexagonal core [52] are presented in round brackets. Irrespective of the core size, the trend of NICS(0) values appears to be the same [58]. The largest aromatic character (*i.e*., largest negative values) was found in 6-atom petals of circulenes [8:6_8_] and [8:(5,6)_4_]; in case of [8:(5,7)_4_], the 5-atom petals show a low aromatic character (compare with the azulene (5,7) molecule). Interestingly, a negative NICS index value was obtained for the 8-core (*i.e*., cyclooctatetraene) of the flower [8:(5,7)_4_] and alternating NICS values (−7.788/−3.863) for the 6-membered petals of circulene [8:6_8_]. The 7-atom petals in the flower [8:(5,7)_4_] show positive NICS values (*i.e*., anti-aromatic character), as expected. The results show that for a saddle-shaped circulene, like the 8-coronene [8:6_8_] the outer aromatic rings (even if they are all 6-rings) are no longer equivalent (by this reason, for 8-coronene, the formula [8:(6,6)_4_]) can be written, in contrast to the 6-coronene [6:6_6_], with all equivalent petals).

Exaltation of magnetic susceptibility was calculated for the discussed octagonal core flowers; negative values of the exaltation of the magnetic susceptibility prove the aromaticity of a molecule, while the opposite means an anti-aromatic character. Data computed according to the above isodesmic reaction schemes, suggested for [8:(5,7)_4_] an anti-aromatic character while for [8:6_8_] and [8:(5,6)_4_], the negative values of exaltation prove for an aromatic character, with [8:6_8_] more aromatic than [8:(5,6)_4_] [58].

#### 2.2.4. HOMA Index (Geometric Criterion)

HOMA index was computed for all the 5-, 6- and 7-atom petals of the octagonal core flowers. The results listed in Table 10 show that the 6-atom petals of [8:(5,6)_4_] flower have the most pronounced local aromatic character. Alternating values for the 6-atom petals of the saddle-shaped circulene [8:6_8_] was observed, as shown in case of NICS(0) values (see Table 9). For the 5- and 7-atom petals, negative values (*i.e*., anti-aromatic character) were reported [58].

**Table 10:** HOMA values, computed for the 8-flowers (optimized at HF/6-311G(d,p) level of theory) [58].

Structure	Core	Petals (5 Atoms)	Petals (6 Atoms)	Petals (7 Atoms)
[8:(5,7)_4_]	−0.811	−0.983	-	−0.414
[8:6_8_]	−0.432	-	0.703/0.335	-
[8:(5,6)_4_]	−0.524	−0.817	0.960	-

#### 2.2.5. Reactivity Descriptors

Besides their use in evaluating the reactivity and regioselectivity of chemical reactions, reactivity descriptors like absolute hardness (η), electrophilicity (ω) and Fukui functions have also been applied to evaluate the aromatic character of molecules [58,78]. The *absolute hardness* (η) is calculated as half of the HOMO-LUMO gap; a harder molecule is associated with an increased stability, so molecules with larger η values are believed to be more stable, thus showing a possible aromatic character. Also, a lower *electrophilicity* ω value can be taken as a proof of aromaticity. Regarding the local reactivity descriptors, the *Fukui functions* computed for an electrophilic attack are good indicators of reactivity of each atom in the studied circulenes, thus a hierarchy of the most electrophilic sites could be established. The above descriptors of reactivity are defined as follows:

$$\text{Absolute hardness }[59]\text{: η}\approx \frac{{\text{ε}}_{\text{LUMO}}-{\text{ε}}_{\text{HOMO}}}{2}$$

$$\text{Electrophilicity index }[59]\text{: ω}=\frac{{\text{μ}}^{2}}{2\text{η}}$$

$$\text{Fukui functions }[60]\text{: }{\text{f}}_{\text{k}}^{\text{α}}={\sum_{\text{μ}\in \text{k}}\left|{\text{c}}_{\text{μ}}^{\text{α}}\right|}^{2}$$

where α = HOMO orbital, with neglection of the overlap integral.

Data for the discussed flowers are given in Table 11; they are in good agreement with the geometric, magnetic and energetic criteria above used to evaluate the aromaticity in the three circulenes [8:(5,7)_4_], [8:6_8_] and [8:(5,6)_4_]. The highest value of the absolute hardness, a measure of the molecular stability, has been obtained for [8:(5,6)_4_] flower, showing the most pronounced aromatic character among the investigated compounds. Conversely, the most electrophilic flower is [8:(5,7)_4_], which is related to its lower aromatic character among the studied molecules.

**Table 11:** Absolute hardness (η) and electrophilicity (ω), (B3LYP/6-311G(d,p)) [58].

Circulene	η (eV)	ω (eV)
[8:(5,7)_4_]	1.07	5.60
[8:6_8_]	1.62	4.08
[8:(5,6)_4_]	1.93	3.38

**Figure 6:**
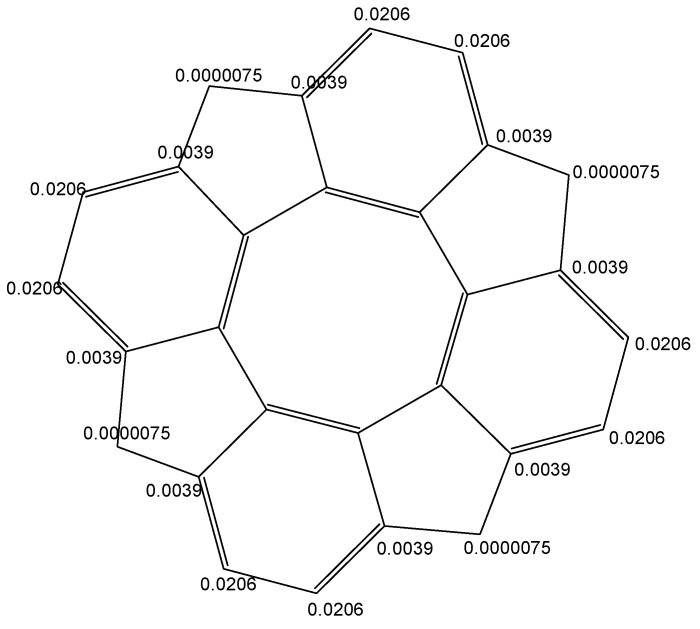
Fukui function (for an electrophilic attack, f^+^) in circulene [8:(5,6)_4_].

The Fukui functions [60] (for an electrophilic attack) have been computed for each carbon atom on the contour of circulenes optimized at (B3LYP/6-311G(d,p) level of theory (Figure 6).

The results should represent an estimation of the place where the electrophilic attack is most likely to occur:

- in circulene [8:(5,7)_4_], the three carbon atoms in the 7-atom petals show different reactivity, suggesting a kind of circular polarization of pi-electrons;
- in flower [8:6_8_], the 6-atom petals show alternating properties, also shown by the values of local aromaticity indices (HOMA and NICS(0));
- circulene [8:(5,6)_4_], the only planar structure, has two equivalents C-atoms on each benzene unit, with a uniform distribution around the molecule.

**Figure 7:**
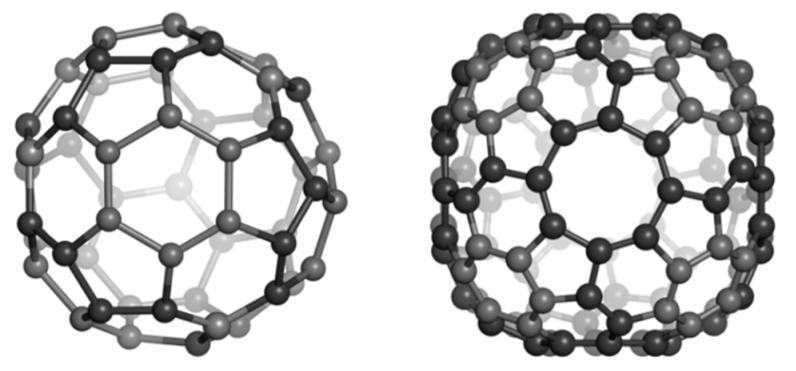
Fullerenes tessellated by 6-Sumanene: C_52_([6:(5,6)_3_]_4_) (**left**) and 8-Sumanene C_120_ ([8:(5,6)_3_]_6_); [6:(5,6)_3_]_8_)) (**right**).

Corroborating the results of different criteria of aromaticity on the three circulenes led to the conclusion that, the most “aromatic” one is the 8-sumanene, [8:(5,6)_4_], in agreement with the fact that tetraoxa[8]circulenes represent real molecules [63]. Following this result, two different fullerenes, bearing 6-sumanene and 8-sumanene patches, have been designed (Figure 7). The values of NICS(0; −1 (inside the cage); +1 (out of the cage)) and HOMA indices, for the 5-, 6-, and 8-rings of the fullerenes in Figure 7 are listed in Table 12.

**Table 12:** Total energy per C atom, E_tot_/C, HL gap, H_f_, NICS and HOMA values for cages C_52_ and C_120_ (optimized at HF/6-31G(d,p)) [58].

Structure/ Substructure	E_tot_/C (au)	HL Gap (eV)	H_f_(kJ/mol)	NICS(−1)	NICS(0)	NICS(+1)	HOMA
C_52_	−37.864	5.317	2095.9				
R[5] petal				−17.783	−2.292	−0.275	−0.093
R[6] petal				−28.663	−15.704	−6.059	0.333
R[6] core				−28.281	−11.372	−2.803	0.187
C_120_	−37.871	6.251	2181.8				
R[5] petal				−12.058	−0.242	−0.711	−0.553
R[6] petal				−16.646	−7.635	−3.589	0.537
R[6] core				−16.746	−7.563	−3.565	0.821
R[8] core				−3.498	5.164	6.196	−1.478

In case of C_120_, the NICS(0) values for the 8-sumanene patch show the same trend as in the planar 8-sumanene. The values NICS(−1), characterizing the inside cage electron density, show more aromatic character (*i.e*., larger negative values) in comparison to the outside cage describing NICS(+1), as expected. Comparing the 6-sumanene patch in the two cages in Figure 7, one can see a more aromatic character of petals vs the core, according to NICS(0) values. The HOMA values follow in general the trend of NICS values, excepting the 6-sumanene patch in C_120_, where the 6-ring core was found more aromatic (*i.e*., more positive value, 0.821) than the 6-ring petal (0.537).

**Figure 8:**
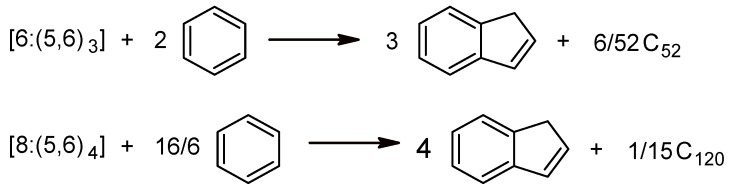
Isodesmic reactions for the enthalpy calculation in fullerenes C_52_ and C_120_.

In order to estimate the stability of the C_52_ and C_120_ cages, two isodesmic reactions have been proposed [58] (Figure 8). The values of H_f_ (Table 12, 4th column) are in good agreement with the ones of the single point computations (E_tot_/C); there are no significant differences between the computed stability of the two structures. However, the HOMO-LUMO gap value is in favor of C_120_ (6.251 for C_120_ *vs*. 5.317 for C_52_).

Concluding, the stability and aromatic character of the three 8-flowers [8:(5,7)_4_], [8:6_8_] and [8:(5,6)_4_] have been investigated by computing NICS and HOMA indices, heats of formation and exaltation of the magnetic susceptibility, as well as local and global reactivity descriptors, in case of an electrophilic attack. The results of the geometric, magnetic and energetic criteria led to the same conclusion: circulene [8:(5,6)_4_] (*i.e*., the planar 8-sumanene [8:(5,6)_4_]) shows the most pronounced aromatic character, while the [8:(5,7)_4_] compound (saddle-shaped, [8:(5,7)_4_]) is the most unstable and is characterized by a weak aromaticity. The trend of NICS and HOMA values was also kept in case of fullerenes bearing 8-sumanene patches.

## 3. Polybenzenes

**Figure 9:**
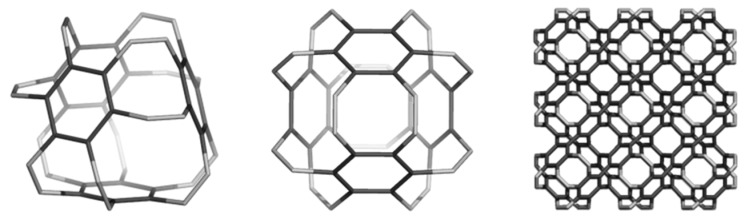
Benzene rings embedded in the *D*-surface; BTA_48 = 6.8^2^ *D* (**left**), the face-centered BTA_48 unit (**middle**) and its diamondoid BTA_*fcc*-network (in a (*k*,*k*,*k*)-domain, *k* = 3, **right**).

**Figure 10:**
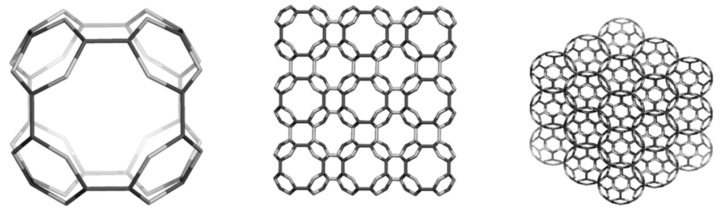
Benzene rings embedded in the *P*-surface; BCZ_48 = 6.8^2^ *P* (**left**), its networks in a cubic (*k*,*k*,*k*)-domain, *k* = 3 (**middle**) and the corner view of this network (**right**).

O’Keeffe *et al*. [79] proposed, about twenty years ago, two 3D networks of benzene: the first one, called 6.8^2^ *D* (also polybenzene, Figure 9), is described to belong to the space group *Pn*3*m* and having the topology of diamond. The second structure (Figure 10) was called 6.8^2^ *P* and it belongs to the space group *Im*3*m*, corresponding to the *P*-type-surface. These networks represent embeddings [80] of the hexagon-patch in the two surfaces of negative curvature, *D* and *P*, respectively.

These triple periodic minimal surfaces (as in the soap foam) can embed networks of covalently bonded *sp*^2^ atoms, called *periodic* *schwarzite* [1,45] in the honor of H. A. Schwarz [81,82] who, in the nineteen century, studied the differential geometry of such surfaces.

The two proposed structures show stability comparable, or even higher, to that of C_60_(*I_h_*) [56,79]. The structure 6.8^2^ *D* was predicted to be insulator while 6.8^2^ *P* metallic. Zeolites [83] and spongy carbon [48,49] represent schwarzite structures.

**Figure 11:**
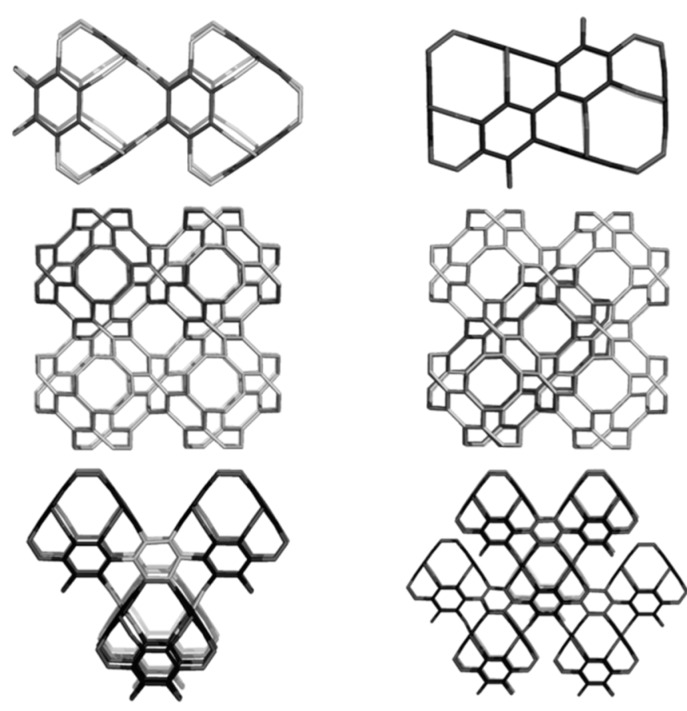
**Top** row: BTA_48 as R(8)-BTA_2dia__88 dimer (**left**) and R(12)-BTA_2den__84 dimer (**right**). **Middle** row: superposition (in black) of R(8)-dimer (**left**) and R(12)-dimer (**right**) on the BTA48 *_fcc_*_222_288 domain of the *fcc*-network. **Bottom** row: dendrimers BTA_5dend__192 (**left**) and BTA_17dend__624 (**right**). In the name of dendrimers, the subscript number indicates the number of repeating units composing the structure while the last number counts C-atoms.

The networks were constructed [84] either by identifying or joining the common faces in the corresponding repeating units, BTA_48 and BCZ_48, respectively (Figure 9 and Figure 10, left). Face identification in case of the armchair-ended, tetrahedral unit BTA_48 is possible either by octagons R(8) or by dodecagons R(12). Identification by R(8) of the BTA_48 units, disposed at the center of the six faces of Cube, leads to the 6.8^2^ *f_cc_*-net (Figure 9, right), with the topology of D_6_-diamond; the corresponding R(8)-dimer we call the “dia-dimer” BTA_2dia__88 (Figure 11, top, left). When R(12) are identified, the resulting oligomers are dendrimers (Figure 11, bottom row) and the R(12)-dimer is named “dendritic dimer” BTA_2den__84 (Figure 11, top, right). Dendrimers, after the second generation, completely superimpose over the BTA48_*f_cc_*-net (Figure 11, middle and bottom rows).

Evaluation of the stability of polybenzenes was performed on finite hydrogen-ended structures (Table 13 and Table 14). Data include the total energy E_tot_, total energy per carbon atom, E_tot_/C-atom, HL gap, strain energy according to Haddon’s POAV theory and HOMA index for the benzene patch R[6]; the reference structure was taken the fullerene C_60_ (*I_h_*). The trend of energy values is similar in HF and DFT approaches. Since no interactions with solvents are of interest here, as DFT approaches can evaluate, for structures of a large number of atoms only HF calculations have been performed.

**Table 13:** Polybenzenes: total energy E_tot_; total energy/C-atom E_tot_/C-atom and HOMO-LUMO HL gap (at Hartree-Fock HF/6-31G(d,p) level of theory), strain (by POAV theory) and HOMA index, with C_60_(*I_h_*) as the reference structure [84].

Structure		No Units	E_tot_ (au)	E_tot_/C (au)	HL Gap (eV)	Strain/C (kcal/mol)	HOMA R[6]
1	BTA_48	1	−1831.484	−38.156	11.285	0.083	0.951
2	BCZ_48	1	−1831.097	−38.148	8.134	3.395	0.989
3	BTA_2dia__88	2	−3355.431	−38.130	10.970	0.074	0.972
4	BTA_2dend__84	2	−3201.679	−38.115	10.895	0.061	0.975
5	BTA_3dend__120	3	−4571.874	−38.099	10.771	0.056	0.978
6	BTA_4dend__156	4	−5942.070	−38.090	10.684	0.054	0.978
7	BTA_5dend__192	5	−7312.265	−38.085	10.594	0.055	0.988
8	C_60_(*I_h_*)	1	−2271.830	−37.864	7.418	8.256	0.493

**Table 14:** Polybenzenes: total energy, E_tot_ (in au), total energy per C-atom, E_tot_/C, and HL gap (in eV), (Hartree-Fock (HF/6-31G(d,p)) and DFT (B3LYP/6-311 + G(d,p)) [85,86].

Structure	E_tot_	E_tot_/C	HL Gap	E_tot_	E_tot_/C	HL Gap
	HF			DFT		
BTA_48	−1831.484	−38.156	11.285	−1843.743	−38.411	5.052
BTA_2ecl__90	−3428.847	−38.098	10.085	−3450.946	−38.344	4.200
BTA_2dia__88	−3355.431	−38.130	10.970	-	-	-
BTA_2int__84	−3201.679	−38.115	10.895	-	-	-
BTA_Cy,5__210	−7986.806	−38.032	9.545	-	-	-
BTZ_24	−915.092	−38.129	8.221	−921.359	−38.390	2.753
BTZ_2ecl__48	−1826.768	−38.058	6.194	−1839.181	−38.316	1.124
BTZ_Cy,5__120	−4558.826	−37.990	7.178	-	-	-
C_60_(I_h_)	−2271.830	−37.864	7.418	−2286.610	−38.110	2.724

Among the structures considered in Table 13, the most stable appears the armchair-ended unit BTA_48, with a tetrahedral embedding of benzene patch (Table 13, entry 1), followed by BTA_2dia__88 (Table 13, entry 3). The third is the dendritic dimer BTA_2dend__84 while the stability of some oligomers (*i.e*., dendrimers) of BTA_48 decreases monotonically with the increase of number of composing units (Table 13, entries 4 to 7) as suggested by E_tot_/C-atom and HL gap. The strain of these dendrimers decreases with the increase in the number of their carbon atoms. This is reflected in the values of HOMA: the benzene patch seems to be little distorted from the ideal planar geometry, with a maximum at the dendrimer with a complete generation, e.g., BTA_5dend__192 (Table 13, entry 7).

**Figure 12:**
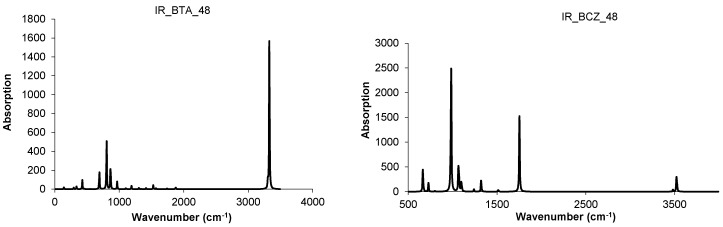
IR spectra of BTA_48 (**left**) and BCZ_48 (**right**) units.

**Figure 13:**
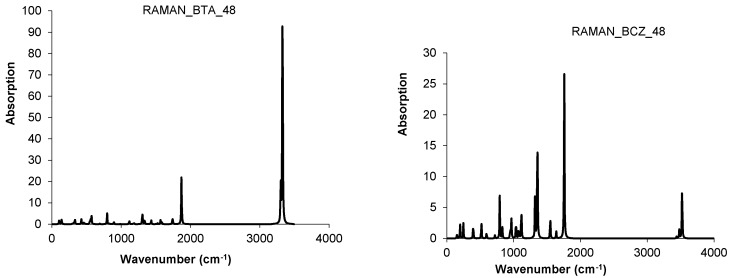
Raman spectra of BTA_48 (**left**) and BCZ_48 (**right**) units.

The BCZ_48 structure (Table 13, entry 2) shows the highest value of HOMA, even the benzene patch is less planar in comparison to the same patch in BTA_48; it is the most strained structure among the all ones in Table 13. It seems that the C_C bond length is not the only parameter reflecting the pi-electron conjugation, as limited by HOMA index. Looking at the data in Table 13, entry 8, the reference fullerene C_60_(*I_h_*) appears the least stable among all the considered structures; recall that it is all-carbon and data cannot be directly compared to those of hydrogen-ended molecules. However, polybenzenes have the total energy per carbon atom close to that of the reference fullerene. For BTA_48, and BCZ_48 the simulated vibrational spectra are given below (Figure 12 and Figure 13) [84].

**Figure 14:**
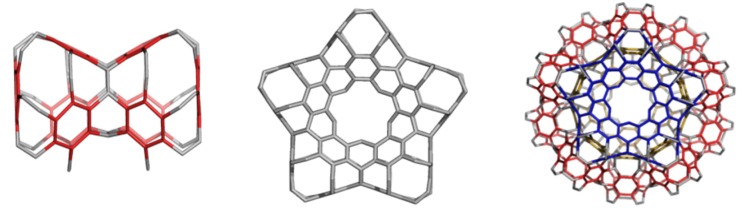
Oligomers of BTA_48: BTA_2ecl__90 (**left**) and BTA_Cy5__210 (**middle**) and the multi-torus BTA_20__780 (**right**).

**Figure 15:**
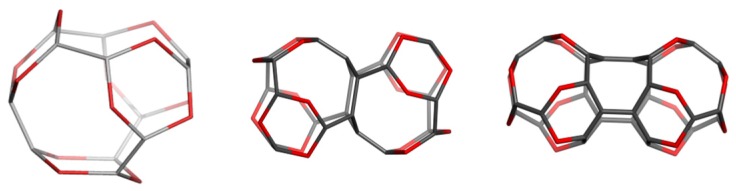
The unit BTZ_24 (**left**) and its dimers: and BTZ_2anti__42 (**middle**) and BTZ_2ecl__48 (**right**).

**Figure 16:**
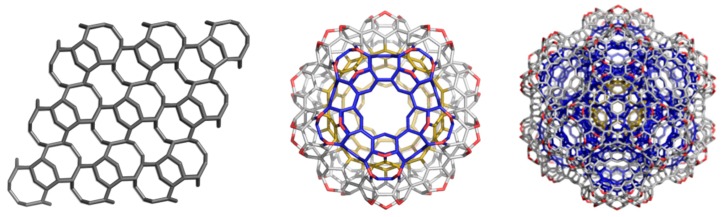
The triple periodic net BTZ24_anti__333_774 (**left**), a multi-torus BTZ_20__480 (**middle**) and its radial hyper-array BTZ_Sph12__3120 (**right**).

There is possible a third dimer, BTA_2ecl__90 (Figure 14, left) in the eclipsed arrangement [85]. It forms a hyper-pentagon, BTA_Cy5__210 (Figure 14, middle), that next self-arranges to the multi-torus BTA_20__780 (Figure 14, right). An even simpler polybenzene is BTZ_24 (Figure 15, left), of which dimers (Figure 15, middle and right) can form a 3-periodic network (Figure 16, left) and multi-tori (Figure 16, middle and right) respectively [85,86].

**Figure 17:**
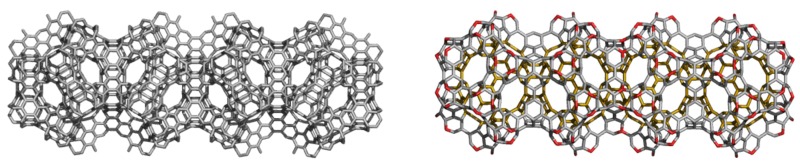
1-periodic rod-like structures: BTA_20__4_2490 (**left**) and BTZ_20__4_1560 (**right**).

The 3-periodic net BTZ24_anti__333 is the well-known uninodal network “*uta*” (point symbol for net: {6.9^2^}; 3-c net), belonging to the space group *Fd-*3*m.* Twelve units of BTZ_20_ (Figure 16, middle) can self-arrange to a quasi-spherical structure, of icosahedral symmetry (Figure 16, right). The units BTA_20_ and BTZ_20_ also can form 1-periodic networks (Figure 17).

**Figure 18:**
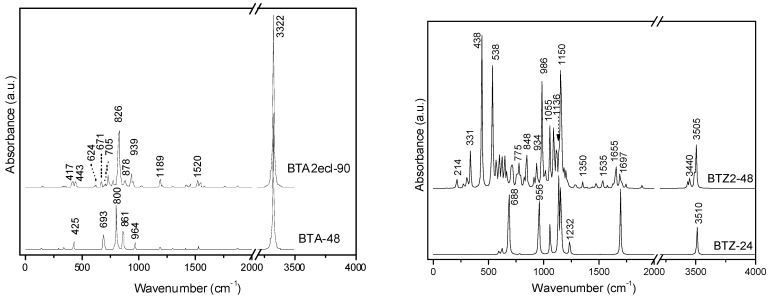
IR spectra of polybenzene monomers and “eclipsed” ecl-dimers: BTA series (**left**) and BTZ series (**right**).

**Figure 19:**
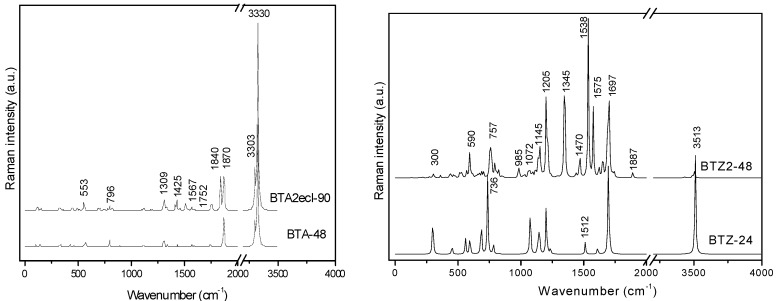
Raman spectra of polybenzene monomers and “eclipsed” ecl-dimers: BTA series (**left**) and BTZ series (**right**).

From Table 14, one can see that, the “armchair” A-structures are more stable than the “zig-zag” Z-structures, according to their total energy per carbon atom and HOMO-LUMO gap values [85,86]. The difference observed between the two series A/Z comes out from the size of the opening ring: 12 in case of A-series and 9 in case of Z-series, even the patch is always a hexagon. The planarity of benzene patch (more planar in case of A-series, than in case of Z-series) will influence both the energetics and vibrational spectra (Figure 18 and Figure 19) of these structures [85].

IR spectra of BTA-48 and BTA_2ecl__90, respectively (Figure 18, left), show in the region 400–720 cm^−1^ the following differences in the absorbance bands: 425 cm^−1^ band splits into 417 and 443 cm^−1^; 693 cm^−^^1^ band splits into 624, 671 and 705 cm^−1^ [87]. This splitting can be interpreted to account for the dimer joining bonds [85]. In the Raman spectra of BTA-48 and BTA_2ecl__90 (Figure 19, left), common features for a benzene-like structure can be identified and an additional Raman signal around 1840 cm^−1^ for the dimer BTA_2ecl__90 as well. In the Z-series, the IR spectrum (Figure 18, right) shows two intense peaks at 438 and 538 cm^−1^ that can be attributed to the dimer bonds. Raman band around 1575 cm^−1^ (Figure 19, right) corresponds to the C–C stretching of the phenyl ring [88,89]. The presence of the vibration modes around 1345 and 1470 cm^−1^ indicate the formation of the dimer [90].

## 4. P-Type Surface Coverings

In the experimental conditions of fullerene synthesis, it is possible that some cages appear spanned, the “open”-faces next suitably joining to each other to eventually form a nanotube. We call such spanned fullerenes “nanotube junctions” [1]. According to their symmetry, we can distinguish tetrahedral, octahedral and icosahedral junctions.

Tetrahedral junctions are particularly interesting due to their similarity with the tetrahedral *sp*^3^ hybridized carbon atom: the valences are now nanotubes while the atom is an opened cage embedded in a surface of genus 2. Recall, an embedding is a representation of a graph on a surface *S* such that no edge-crossings occur [80]. Genus is the number of handles to be attached to the sphere to make it homeomorphic to the surface on which a graph was embedded, or the number of connections of a given surface (the reader can find more information about structures of high genera, in [1,45]). As the single C-atom, a tetrapodal junction can be used to build various nanostructures such as diamondoids and multi-tori. Octahedral junctions (of genus g = 3) appear in zeolites, of which associated graphs are embedded in the P-type surface. Icosahedral junctions are also possible, as they appear in icosahedral multi-tori [91]. Zeolites [83] are natural or synthetic alumino-silicates with an open three-dimensional crystal structure. Zeolites are micro-porous solids used as “molecular sieves”.

### 4.1. Sumanene Including Structures

**Figure 20:**
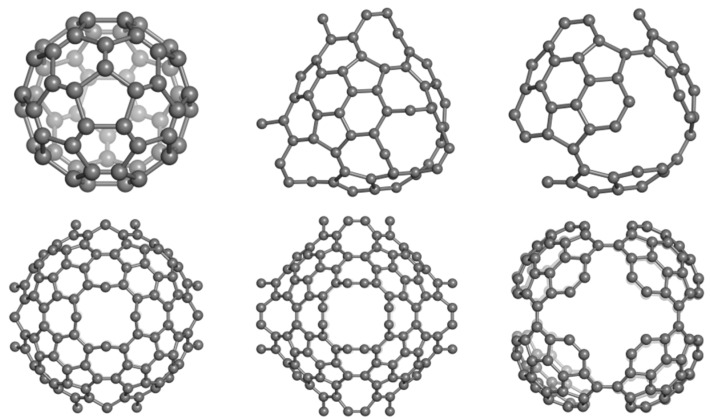
**Top** row: C_60_(*I_h_*) (**left**), Sum_TA_108 (**middle**); Sum_T_84 (**right**). **Bottom** row: Sum_CZ_192 (**left**); Sum_CA_216 (**middle**); Sum_S_2_LeX_168 (**right**).

Sumanene can be used as a primary real molecule in the synthesis of some structural units: Sum_T_A_108, Sum_T_84, Sum_CZ_192, Sum_CA_216, and Sum_S_2_LeX_168 (Figure 20), that can next compose more complex nanostructures, e.g. ordered schwarzites, embedded in the P-surface [92].

**Figure 21:**
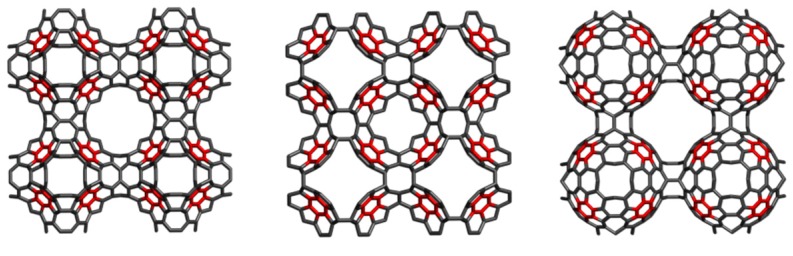
P-networks designed by Sum_CA_216 (**left**), Sum_S_2_LeX_168 (**middle**) and Sum_CZ_192 (**right**); *k* = 2.

Hypothetical crystal carbon networks can be built up from the units listed in Figure 20, either by identifying two opposite open faces (Figure 21, left), or by joining the opposite atoms (Figure 21, middle and right), by the aid of Nano Studio software [93], that also enables their embedding in the P-type surface [1,2]; these networks belong to the space group *Pn*3*m*. Stability of the H-ended structures bearing the sumanene patch (Figure 20) was evaluated; Table 15 lists the energetic data, obtained after optimization at Hartree-Fock (HF) level of theory [92].

From Table 15, it is clear that such molecular structures show values of E_tot_/C comparable to that of C_60_(*I_h_*) reference structure; Sum_T_84 and Sum_S_2_LeX_168 are the most simple and stable units, possible candidates for laboratory synthesis.

**Table 15:** Total energy, E_tot_ (in au), total energy per C-atom and HL Gap (in eV), (at HF/6-31G(d,p) level of theory) for H-ended sumanene-patched structures in Figure 20 [92].

Structure	No. C	E_tot_	E_tot_/C	HL Gap
C_60_(*I_h_*)	60	−2271.830	−37.864	7.418
Sum_TA_108	108	−4103.136	−37.992	7.259
Sum_T_84	84	−3194.384	−38.028	7.562
Sum_CZ_192	192	−7298.367	−38.012	6.044
Sum_CA_216	216	−8206.401	−37.993	6.442
Sum_S_2_LeX_168	168	−6389.018	−38.030	6.637

### 4.2. Spanned Cages Patched by Hexagons Only

**Figure 22:**
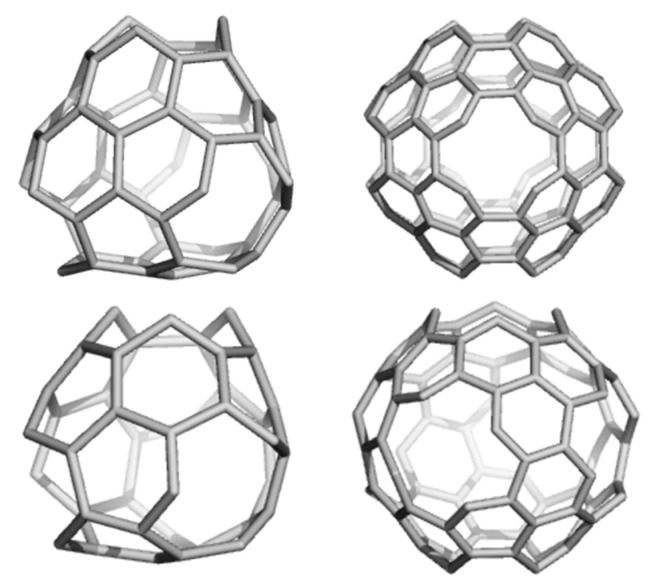
T_3HexZ_52 (**top**, **left**), C_3HexZ_104 (**top**, **right**), T_3HextwZ_40 (**bottom**, **left**) and C_3HextwZ_80 (**bottom**, **right**).

A covering by a single type polygon is called a Platonic tessellation [1]. The units in Figure 22 were designed [94,95] either by using symmetry in embedding the triple hexagon patches (Figure 22, top row) or by applying map operations *Op*_2*a*_(*S*_2_(M)); M = tetrahedron T, or cube C (Figure 22, bottom row). Observe the twisted/chiral appearance of these last units, about 90 degree in case of C_3HextwZ_80. More about the map operations can be found in [96,97,98].

**Figure 23:**
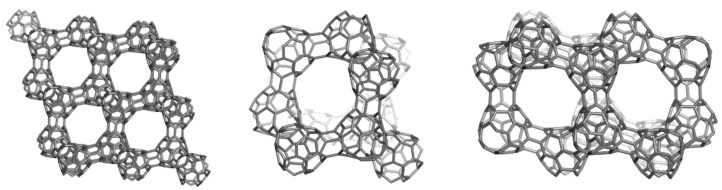
Diamondoid network T_3HextwZ_(2,2,2)_1760 (**left**) built from the unit T_3HextwZ_40, and its substructures Ada_400 (**middle**) and Dia_560 (**right**).

The unit T_3HexZ_52 provides an “eclipsed” dimer, which can self-arrange to a hyper-pentagon, the join of 12 such hyper-faces leading to a multi-torus T_3HexZ20_1040 (not seen) of icosahedral symmetry. In the opposite, T_3HextwZ_40 forms an “intercalated” dimer, next leading to a hyper-hexagon, which can arrange in a diamondoid network [95], as shown in Figure 23.

**Figure 24:**
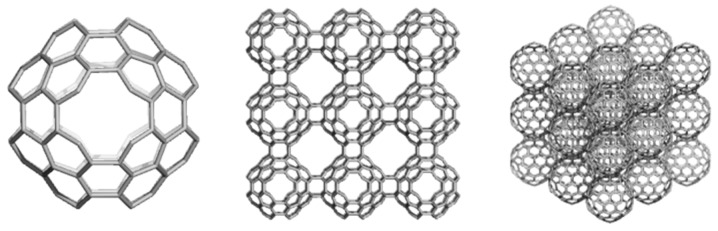
C_3HexZ_104, *g* = 3 (**left**), forms a P-type crystal network C_3HexZ_(3,3,3)_2808 (**middle**); the same network, shown in the corner view (**right**).

The unit C_3HexZ_104, containing triple hexagon 3*f*_6_ patches, forms a 3-periodic lattice, embedded in the P-surface (Figure 24). The C_3HexZ network is a new one, designed by TOPO Group Cluj, a 4-nodal net of the *Pm*-3*m* group. It has the point symbol for net: {6.8^2^}3{6^2^.8}6{6^3^}4 and vertex symbol [6.6.6] [6.6.6] [6.6.8] [6.8.8]. Stability evaluation was done on H-ended molecules, optimized at Hartree-Fock level of theory; data are listed in Table 16. One can see that, the HOMO-LUMO gap (calculated at HF level of theory) is the highest for the reference fullerene C_60_(*I_h_*) (Table 16, entry 5), however, E_tot_/C-atom, is favorable to the “twisted” junctions (Table 16, entries 3 and 4), even the strain of these structures, calculated by POAV theory, is higher than for the non-twisted ones. The strain is lower for the octahedral junctions (Table 16, entries 2 and 4), as expected for structures with larger “open” faces. The HOMA index values follow the trend of strain data [94,95]. Similarly, the Kekulé structure count [11] is in favor of octahedral junctions.

**Table 16:** Triple hexagon-patched (H-ended) structures: total energy per C-atom, E_tot_/C, and HL gap (at (HF/6-31G**); strain energy per C-atom, (by POAV1); HOMA aromaticity index and Kekulé structure count [94,95].

Structure		E_tot_/C (au)	HL Gap (eV)	Strain/C (kcal/mol)	HOMA Patch	Kekulé Count
1	T_3HexZ_52	−37.986	6.140	5.435	−0.131	972
2	C_3HexZ_104	−37.999	5.342	2.329	0.258	944784
3	T_3HextwZ_40	−38.021	6.681	5.799	−0.583	72
4	C_3HextwZ_80	−38.036	6.050	2.551	−0.020	11025
5	C_60_(*I_h_*)	−37.864	7.418	8.256	0.493	12500

**Figure 25:**
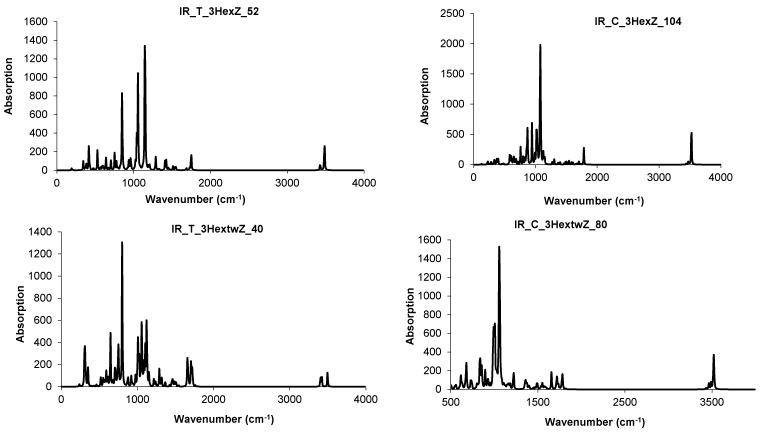
IR spectra for some 3Hex- (**top** row) and 3Hextw- (**bottom** row) patched junctions.

**Figure 26:**
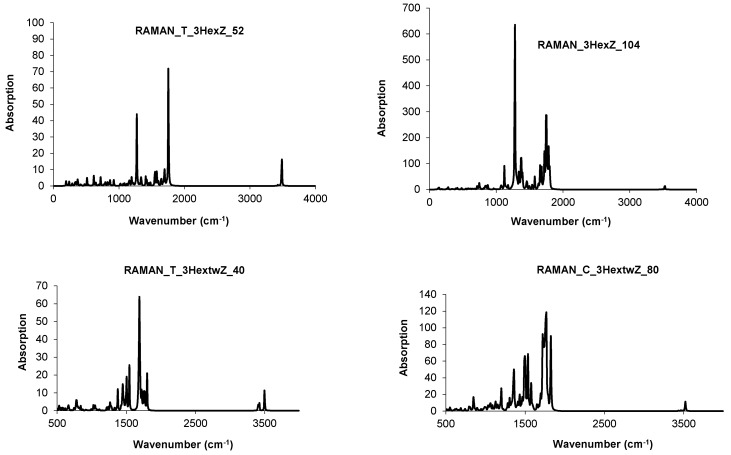
Raman spectra for some 3Hex- (**top** row) and 3Hextw- (**bottom** row) patched junctions.

Resuming, the junctions patched by triple hexagons show several stability parameters close to those of the reference C_60_(*I_h_*) fullerene. In supporting the idea that various nanotube junctions could appear in real experiments, we simulated the vibrational spectra of these junctions (Figure 25 and Figure 26). These spectra show clear differences between the two different embeddings (in tetrahedra and cubes, respectively) and also between twisted and non-twisted ones [94,95].

### 4.3. Nanotube Junctions Patched by Heptagons Only

**Figure 27:**
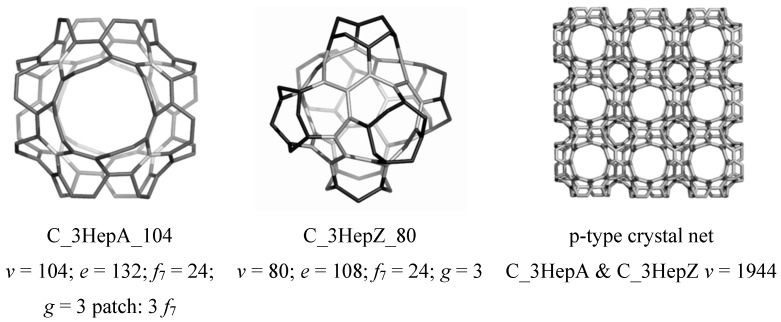
Triple-heptagon patched units (**left** & **middle**) and the corresponding p-type network (**right**): the large hollows correspond to C_3HepA_104 (left) while the small ones to C_3HepZ_80 (middle).

The units obtained by applying the septupling *S_k_*, *k* = 1,2 map operations on the cube C, can form translatable crystal networks, as illustrated in Figure 27. This is the already known *kgn* network, a 3-nodal one belonging to the group *P*432, with the point symbol for the net (7^3^) and the vertex symbol [7.7.7] [7.7.7] [7.7.7]. It is related to the well-known Klein graph [1]. One can see the large hollows represent C_3HepA_104 (Figure 27, left, designed by *Op*_2*a*_(*S*_2_(C)) while the small hollows come from C_3HepZ_80 (Figure 27, middle, designed by *Op*(*S*_1_(C)) [94].

A stability test was done on H-ended molecules, optimized both at the HF and DFT levels of theory; data are listed in Table 17. From these data, one can see that the highest value, among the considered structures, for the total energy per C-atom was provided by the Platonic all-pentagon C_20_ smallest fullerene (Table 17, entries 1, 5); this is probably due to its huge strain, higher 3.3 times than that of C_60_(*I_h_*) (Table 17, entries 4, 8). The pyramidalization of *sp*^2^ C-atoms, as evaluated by the Haddon’s POAV theory [57], is related to the strain energy appearing in the graphite sheet when it is forced to embed in the sphere (*i.e*., closed fullerenes) or in other surfaces (the case of open fullerenes, herein studied). An increased strain value suggests an increased percent of *sp*^3^-hybridized C-atom, reflected in the C-C bond length (see Table 17, the last two columns). The bond-length values suggest an extent of alternant double/single C=C/C-C bonds; none of the studied structures is significantly aromatic, in agreement with their HOMA index of aromaticity, that shows values less than 0.5 (1 being the reference benzene molecule). Since the pyramidalization angles can be calculated either on closed or open (end-hydrogenated) fullerenes, the strain energy data in closed/open structures can be directly compared [94].

**Table 17:** Triple heptagon-patched (H-ended) structures: energies (total energy per carbon atom E_tot_/C, HLGap, (at HF/6-31G** and B3LYP/6-311+G**, respectively); POAV strain energy per C-atom); HOMA index of aromaticity, Kekulé structure count, extreme C-C bond length, and averaged bond length, in Ang [94].

No.	Structure	E_tot_/C (au)	HLGap (eV)	Strain (kcal/mol)	C_60_(*I_h_*) Relat. Strain	HOMA	Kekulé Count	C-C Min/Max	C-C Average
HF									
1	C_20_	−37.828	6.819	27.250	3.301	0.194	36	1.405 1.472	1.439
2	T_3HepA_52	−38.126	7.057	0.410	0.050	−0.951	75	1.312 1.500	1.406
3	C_3HepA_104	−38.127	6.942	0.240	0.029	−0.842	3600	1.313 1.503	1.408
4	C_60_(*I_h_*)	−37.864	7.418	8.256	1.000	0.343	12500	1.373 1.449	1.411
DFT									
5	C_20_	−38.080	1.912	26.730	3.238	−0.361	36	1.398 1.537	1.467
6	T_3HepA_52	−38.382	1.413	0.410	0.050	−0.951	75	1.312 1.500	1.406
7	C_3HepA_104	−38.376	1.354	0.192	0.023	−0.244	3600	1.334 1.482	1.408
8	C_60_(*I_h_*)	−38.110	2.724	8.256	1.000	0.299	12,500	1.392 1.452	1.422

The lowest strained structures in Table 17 are the Platonic all-heptagon open units; this is due to the patch 3Hep = 3*f*_7_, either as the free molecule C_16__3*f*_7_ (strain (HF): 0.044; strain(DFT): 0.089 kcal/mol) or included in these open fullerenes (Table 17, entries 2,3; 6,7). The strain value, in these two units, is two orders of magnitude lower than that in C_60_(*I_h_*). The 3*f*_7_ patch behaves quite the same, irrespective of embedding: tetrahedron, T_3HepA_52 (g = 2, entries 2, 6) or cube, C_3HepA_104 (g = 3, entries 3, 7). The averaged C-C bond length values (Table 17, last two columns) show the lowest value for these all heptagon open fullerenes (1.406 and 1.408 Ang, respectively) supporting their lowest strain values. They also show the lowest values of E_tot_/C-atom, predicting a good stability of these yet hypothetical molecular structures.

HOMA geometric index values collected in Table 17 are irrelevant, suggesting a rather anti-aromatic character for these relaxed structures. Similarly, the values of Kekulé structure count, related to the conjugation of pi-electrons, suggest this phenomenon is less important in the studied structures, while the strain originating in the sigma bond skeleton is a dominant feature.

**Figure 28:**
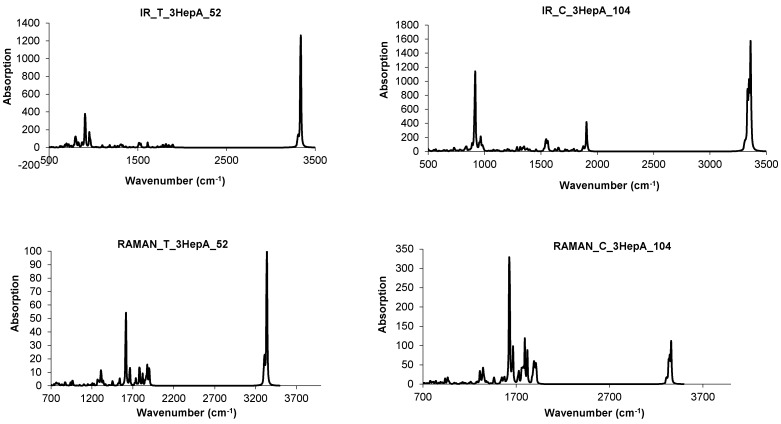
IR and Raman spectra for the 3Hep-patched junctions.

**Figure 29:**
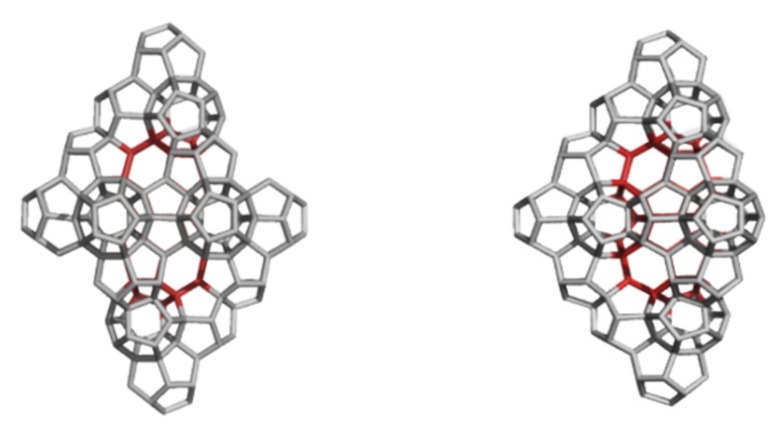

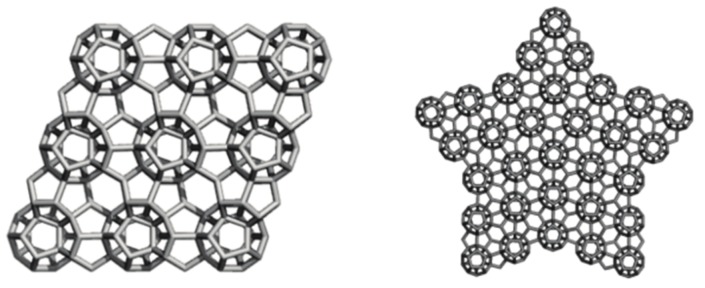
Diamond D_5_ and two of its allotropes: D_5__dia_anti_306 (**top**, **left**); D_5__dia_syn_270 (**top**, **right**); D_5__anti_333_860 (**bottom**, **left**) and D_5__syn_2028_531s_1185 (**bottom**, **right**).

The simulated IR and Raman spectra (Figure 28 and Figure 29) for these triple heptagon-patched open structures show all vibrations with no imaginary values, proving the optimized structures represent global minima [94]. Data collected in Table 18 show that the 3*f*_7_–patch has its fingerprint in IR spectrum; the peaks in bold-italic represent the most intense vibrations, useful as a marker, for an eventual experimental (quick) identification of these structures.

**Table 18:** Spectral data for the triple heptagon-patched structures [94] *.

Structure	T-3HepA_52		C-3HepA_104	
spectrum	IR	Raman	IR	Raman
cm^−1^	903	**1617**	**917**	1617
	**3332**	**3332**	**3332**	*1624*
	*3339*	*3339*	3339	**1631**
	3346	3346	**3346**	
			*3360*	3360

*: normal font = medium peak; bold = intense peak; bold&italic = marker peak.

## 5. Allotropes of D_5_ as Hyper-Graphenes

A crystal structure with pentagon/hexagon rings, of which 90% pentagons, we call diamond D_5_, is known as the clathrate II structure, or *mtn*, a 3-periodic, 3-nodal net, of point symbol net: {5^5.6}12{5^6}5 and 2[5^12^]; [5^12^.6^4^] tiling and belonging to the space group: *Fd-*3*m*. The clathrate II structure exists in the synthetic zeolite ZSM-39 [83,99], in silica [100] and in germanium allotrope Ge(*cF*136) [101,102] as real substances.

Substructures of D_5_ are related to the classical D_6_ diamond [3]. An adamantane-like structure D_5__ada can form two diamantane-like D_5__dia forms (Figure 29, top row). Next, D_5__dia_anti substructure will form a 3-periodic crystal network (Figure 29, bottom, left) while D_5__dia_syn will arrange into a star-like quasi-crystal (Figure 29, bottom, right).

**Figure 30:**
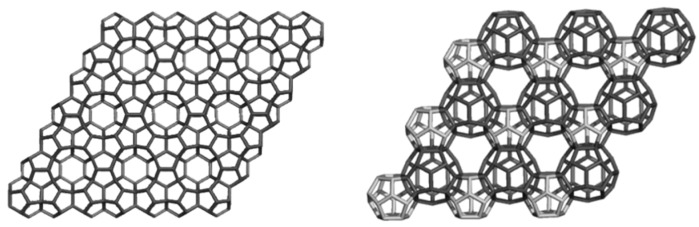
“Exfoliation” of the hypothetical diamond D_5_ leading to hyper-graphenes: C_20_Hex_333_506 (a planar pure hexagonal C_20_ hyper-graphene—**left**); (C_20_C_28_)Hex_331_327 (a sheet of alternating C_20_/C_28_ armchair hyper-hexagonal unit—**right**).

The small fullerenes C_20_ and C_28_, filling the space in the frame of D_5_, can provide, by “exfoliation”, hyper-graphenes (Diudea MV, 2013), either as single-cages (Figure 30, left) or mixed ones [103] (Figure 30, right).

**Figure 31:**
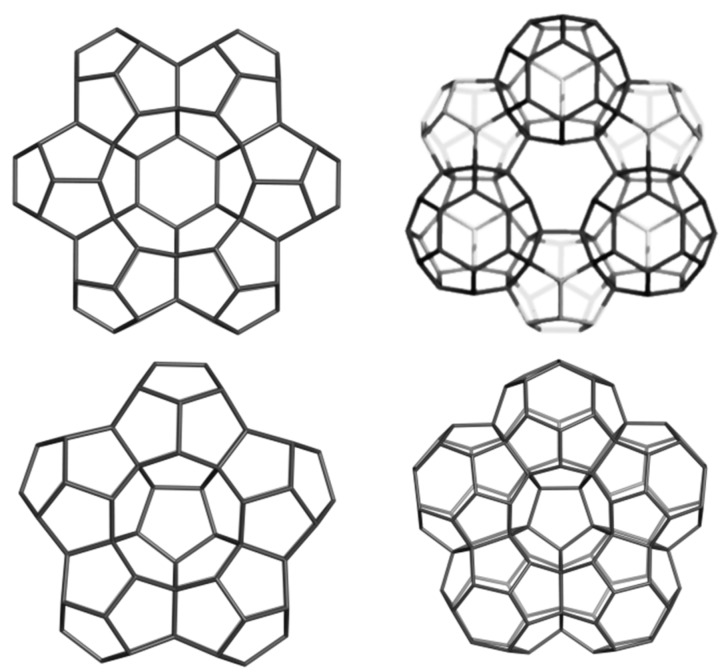
Substructures of C_20_/C_28_ hyper-graphenes. **Top** row: (C_20_)_6__90 (**left**); (C_20_C_28_)_3__114 (**right**). **Bottom** row: (C_20_)_5__75 (**left**); (C_28_)_5__110 (**left**).

**Figure 32:**
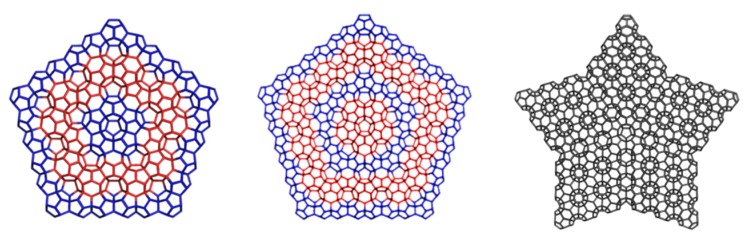
Five-fold symmetry hyper-graphene domains: C_20_-centered (D_5__2028_541p_660, **left**); C_28_-centered (D_5__2820_541p_1170, **middle**) and a D_5_-quasicrystal (D_5__2820_533s_5060, **right**).

The corresponding substructures of the hyper-graphenes in Figure 30 are illustrated in Figure 31 (top row). Alternating C_20_/C_28_ hyper-graphene domains with five-fold symmetry (Figure 32) can result by sectioning a quasi-crystal (Figure 32, right) by an electron beam [103]. Pentagonal hyper-rings appearing in the core of these stars are illustrated in Figure 31, bottom.

Data for the above hyper-graphenes are collected in Table 19 [103]. Data for some small fullerenes and corresponding 5-fold and 6-fold hyper-cycles [104] are presented in Table 20.

**Table 19:** Energetic data (at DFTB level of theory) for some (hydrogenated) C_20_-based hyper-graphenes HG; reference structure: C_60_H_60_ [103].

C_20__Hyper-Graphene	C atoms	E_tot_ (au)	E_tot_/C-atom (au)	HL gap (eV)
C_20_HG_11_90H_60_ = (C_20_)_6__90H_60_	90	−178.393	−1.982	8.992
C_20_HG_22_252H_136_	252	−487.798	−1.936	8.447
C_20_HG_44_780H_360_	780	−1487.55	−1.907	8.191
C_20_HGCor_621_384H_192_	384	−737.736	−1.921	8.307
C_20_HGCor_631_882H_396_	882	−1678.02	−1.903	8.155
C_60_H_60_	60	−125.584	−2.093	10.412

**Table 20:** Energetic (DFTB) data for some small fullerenes and hyper-cycles [104].

Structure	C Atoms	E_tot_ (au)	E_tot_/C-Atom (au)	HL Gap (eV)
C_60_(*I_h_*)	60	−102.185	−1.703	1.930
C_20_	20	−33.429	−1.671	0.731
C_24_	24	−40.142	−1.673	1.667
C_28_	28	−47.101	−1.682	0.351
(C_24_)_5__90	90	−152.998	−1.700	1.634
(C_20_C_28_)_3__114	114	−192.488	−1.688	0.166
(C_20_)_5__75H_50_	75	−146.956	−1.959	9.969
(C_24_)_5__90H_60_	90	−175.282	−1.948	9.103
(C_28_)_5__110H_80_	110	−220.185	−2.002	9.270
(C_20_)_6__90H_60_	90	−178.393	−1.982	8.992
(C_20_C_28_)_3__114H_84_	114	−226.346	−1.985	10.278
C_60_H_60_	60	−125.584	−2.093	10.412
C_20_H_20_	20	−41.659	−2.083	12.295
C_24_H_24_	24	−49.752	−2.073	12.247
C_28_H_28_	28	−58.301	−2.082	12.384

**Figure 33:**
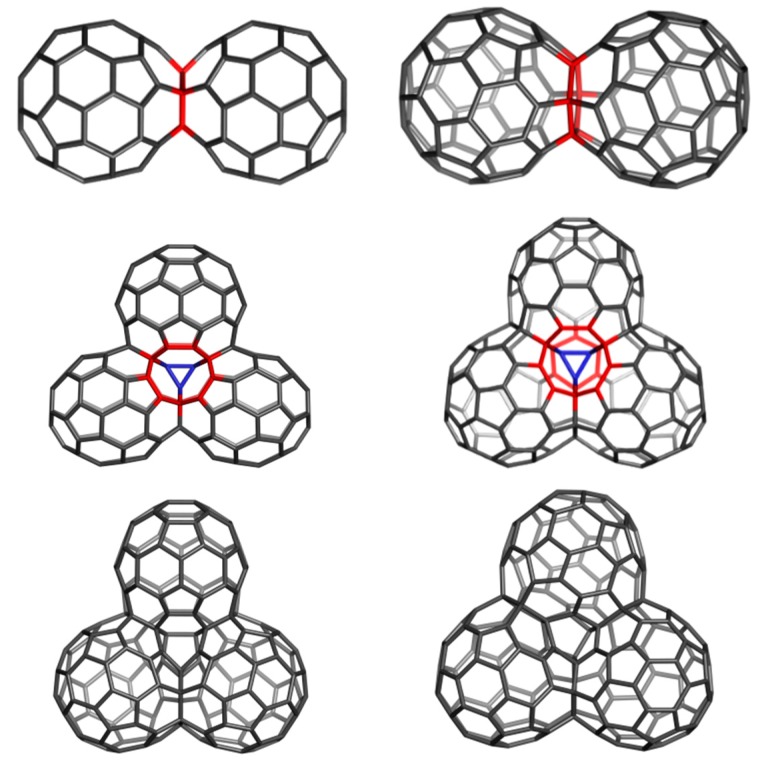
C_60_(*I_h_*) oligomers. **Top** row: C_60_P2J5_115 (**left**) and C_60_P2J6_114 (**right**); **Middle** row: C_60_P3J555_165 (**left**) and C_60_P3J666_162 (**right**); **Bottom** row: C_60_P3J556_164 (**left**) and C_60_P3J566_163 (**right**).

**Figure 34:**
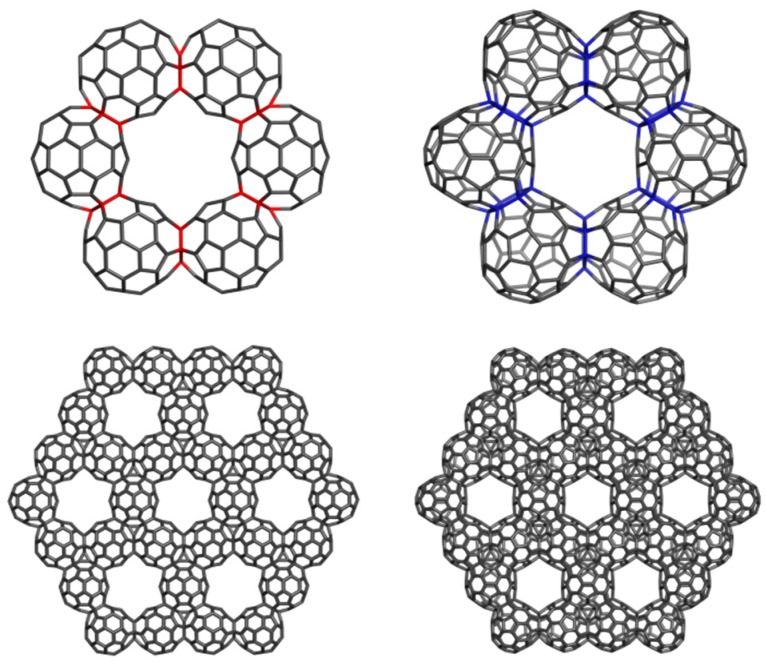
Hyper-graphenes. **Top** row: Hex(C_60_J5)_330 (**left**); Hex(C_60_J6)_324 (**right**); **Bottom** row: (Le(Cor(C_20_))J5_1560 (**left**); Cor(C_60_)J6_1512 (**right**).

A hyper-graphene could be conceived to appear when a thin layer of C_60_(*I_h_*) is deposited on a (plane) surface. The polymerization process can start with a [2+2] cyclo-adduct but this is just the beginning of a more complex process, next following the coalescence of quasi-spherical units of C_60_(*I_h_*) to form oligomers and finally a polymer (Figure 33 and Figure 34); Table 21 supports this idea [104].

**Table 21:** Energetic (DFTB) data for some oligomers of C_60_(*I_h_*) [104].

Structure	C Atoms	E_tot_ (au)	E_tot_/C	HL Gap(eV)
C_60_(*I_h_*)	60	−102.185	−1.703	**1.930**
C_60_P2J5_115	115	−195.708	−1.702	**2.044**
C_60_P2J6_114	114	−194.183	−1.703	**1.444**
C_60_P3J555_165	165	−280.787	−1.702	0.608
C_60_P3J556_164	164	−281.658	−1.717	0.333
C_60_P3_J566_163	163	−280.238	−1.719	0.391
C_60_P3J666_162	163	−278.935	−1.722	**1.481**
Hex(C_60_J5)_330	330	−567.506	−1.710	0.179
Hex(C_60_J6)_324	324	−557.737	−1.721	**1.255**
Le(Cor(C_20_))J5_165_1560	1560	−2652.462	−1.700	0.021
Cor(C_60_)J6_162_1512	1512	−2603.270	−1.722	**1.095**

Let us detail the structures participating to such a process. Two dimers with joint face for C_60_(*I_h_*) units can be designed (Figure 33, top): C_60_P2J5_115 (J5 meaning a pentagon identification) and C_60_P2J6_114 (J6 representing a hexagon identification). These two dimers have the total energy per C atoms comparable to C_60_(*I_h_*); the HOMO-LUMO gap of “J5”-dimer is larger than that of “J6”-dimer (even “J5” dimer has no Kekulé structures).

Next, among the four trimers (Figure 33, middle and bottom) the most stable (see the total energy per carbon atom and gap values in Table 22) appears to be C_60_P3J666_162. The two highly distorted trimers (C_60_P3J556_164 and C_60_P3J566_163) are less stable and further will not be considered.

The “J555” trimer C_60_P3J555_165 shows a lower gap probably because no Kekulé structure can be written. This could be not an argument since the “J5”-dimer also does not admit a Kekulé structure. At a higher number of carbon atoms (see structures in Figure 33) the Kekulé structures are possible for the both J-type polymers while the J6-type joining appear the most stable.

**Table 22:** Comparative data for some small structures involved in hyper-graphenes at Hartree-Fock (HF/6-31G(d,p)), DFT (B3LYP/6-31G(d,p)) and DFTB levels of theory [104].

Structure	C Atoms	Theory	E_tot_ (au)	E_tot_/C (au)	Gap(eV)
C_20__Cy5J5_75	75	HF	−2838.062	−37.841	4.158
C_20__Cy6J5_90	90		−3405.751	−37.842	5.990
C_28__Cy5J6_110	110		−4163.361	−37.849	5.533
C_28__Cy6J6_132	132		−4996.056	−37.849	5.421
C_60_(*I_h_*)_HF	60		−2271.830	−37.864	7.418
C_60_P2J5_115	115		−4354.333	−37.864	7.597
C_60_P2J6_114	114		−4316.491	−37.864	6.270
C_20__Cy5J5_75	75	DFT	−2856.161	−38.082	0.600
C_20__Cy6J5_90	90		−3427.462	−38.083	0.900
C_28__Cy5J6_110	110		−4189.837	−38.089	1.072
C_28__Cy6J6_132	132		−5027.845	−38.090	1.059
C_60_(*I_h_*)_DFT	60		−2286.174	−38.103	2.760
C_60_P2J5_115	115		−4381.797	−38.103	2.907
C_60_P2J6_114	114		−4343.730	−38.103	1.908
C_20__Cy5J5_75	75	DFTB	−126.324	−1.684	0.113
C_20__Cy6J5_90	90		−151.694	−1.684	0.195
C_28__Cy5J6_110	110		−185.928	−1.690	0.006
C_28__Cy6J6_132	132		−223.166	−1.691	0.035
C_60_(*I_h_*)_DFTB	60		−102.185	−1.703	1.930
C_60_P2J5_115	115		−195.708	−1.702	2.044
C_60_P2J6_114	114		−194.183	−1.703	1.444

It is no matter which one of the oligomers will be formed, the hyper-graphene has a good chance (see the boldface HL gap values in Table 21) to exist as areal structure. Note the hyper-graphene Le(Cor(C_20_))J5_165_1560 was designed by applying the leapfrog *Le* map operation [96,97,98] on the coronene-like structure made from the C_20_ smallest fullerene. The hyper-graphene Cor(C_60_)J6_162_1512 was designed by identifying the hyper-hexagons Hex(C_60_J6)_324.

Comparative computations, at HF, DFT and DFTB levels of theory, have been done on small substructures (Table 22). One can see that, in general, the ordering in the three approaches is preserved, of course with some exceptions. The main drawback of DFTB is the underestimation of the gap values in case of *sp*^2^ carbon-only structures (see Table 22). However, DFTB is useful in ordering series of rather large carbon nanostructures [104].

## 6. Computational Details

The geometries of the polycyclic hydrocarbon molecules have been optimized at the HF/6-31G(d) and B3LYP/6-31G(d) level of theory, with the Gaussian 09 suite of programs [105]. The polybenzenes and fullerenes were optimized at the Hartree-Fock HF (HF/6-31G**) and DFT (B3LYP/6-311+G**) levels of theory, while the vibrational spectra (IR and Raman) were performed on the HF optimized structures. Sumanene fullerenes and carbon nanotube junctions were optimized at the Hartree-Fock HF (HF/6-31G**) level only.

Geometry optimization of the circulenes and cyclic compounds appearing in the isodesmic reaction schemes (benzene, naphthalene, cyclooctatetraene, indene, phenanthrene, azulene, acenaphthylene, fluorine and coronene) has been performed at HF/6-311 G(d,p) level of theory. No imaginary frequencies were obtained. In order to compute the enthalpies of formation of the 6-flowers [6:6_6_], [6:(5,7)3] and [6:(5,6)3], the reaction energy for all the six isodesmic schemes was computed using the Equation derived from the Hess law:

$${\text{E}}_{\text{reaction}}=\left(\sum {\text{E}}_{\text{P}}-\sum {\text{E}}_{\text{R}}\right)+\left(\sum \text{ZP}{\text{E}}_{\text{P}}-\sum \text{ZP}{\text{E}}_{\text{R}}\right)+\left(\sum \text{T}{\text{C}}_{\text{P}}-\sum \text{T}{\text{C}}_{\text{R}}\right)$$

where E_P_, E_R_ are the total energies of products and reactants, ZPEP, ZPER are the zero point corrections while TC_P_, TC_R_ represent the thermal corrections.

Suppose the isodesmic reactions correspond to the reaction:

Circulene + PAH_1_ → PAH_2_ + PAH_3_ + PAH_4_

Then, the heat of formation $\Delta {\text{H}}_{\text{f}}$ for a given circulene can be written as:

$$\Delta {\text{H}}_{\text{f}}(\text{Circulene})=\Delta {\text{H}}_{\text{f}}(\text{PA}{\text{H}}_{2})+\Delta {\text{H}}_{\text{f}}(\text{PA}{\text{H}}_{3})+\Delta {\text{H}}_{\text{f}}(\text{PA}{\text{H}}_{4})-\Delta {\text{H}}_{\text{f}}(\text{PA}{\text{H}}_{1})-{\text{E}}_{\text{reaction}}$$

The experimental heats of formation of the PAHs (e.g., benzene, naphthalene, cyclooctatetraene, indene, phenanthrene, azulene, acenaphthylene and fluorene) were taken from ref. [61]. For computing the exaltation of magnetic susceptibility of the 8-flowers [8:(5,7)_4_], [8:6_8_] and [8:(5,6)_4_], the changes (ΔΛ) in the magnetic susceptibility for all the six isodesmic schemes have been computed using the equation: $\text{ΔΛ}={\text{χ}}_{\text{P}}-{\text{χ}}_{\text{R}}$ , where χ_P_, χ_R_ represent the magnetic susceptibilities computed at B3LYP/6-311G(d,p) level of theory. Next, the exaltation of magnetic susceptibility of the corresponding circulene can be written as:

$$\text{Λ}(\text{Circulene})=\text{Λ}(\text{PA}{\text{H}}_{2})+\text{Λ}(\text{PA}{\text{H}}_{3})+\text{Λ}(\text{PA}{\text{H}}_{4})-\text{Λ}(\text{PA}{\text{H}}_{1})-\text{ΔΛ}$$

The magnetic susceptibilities of the needed compounds (namely: benzene, naphthalene, cyclooctatetraene, indene, phenanthrene, azulene, acenaphthylene, fluorine and coronene) were taken from ref. [106].

NICS indices [51] were calculated (using the GIAO method [107]) at the ring centers (NICS(0)) and at 1 Å above and under the centers (NICS(+1), NICS(−1)). All data have been computed by the Gaussian 09 suite of programs [105]. HOMA indices [38,39,40,108] and strain energy, according to POAV1 Haddon’s theory [56,57] were computed with JSChem [109] program. Operations on maps were made by CVNET program [110] while the network building was calculated with the Nano Studio software package [111].

In supporting the choice of the above mentioned levels of theory and basis sets, first is should be said that we kept in mind both the size of molecules to be modeled and the computational cost. In recent years, the B3LYP method has gained immense popularity: at a relatively low computing cost this method gives satisfactory results in the description of structural and electronic properties of molecules [112,113,114]. Published results of some theoretical studies have shown that B3LYP method is suitable in describing aromatic systems, thus being a good alternative to the costly MP2 method [115,116]. In evaluating the aromaticity of molecules, the criterion based on the resonance energy (RE) gives a good inside on the stability of the aromatic compounds.

Also, the isodesmic and homodesmic reactions, using some experimental thermodynamic data, along with computed data (by DFT methods, e.g., B3LYP/6-311+G**) can bring a light on the stability of molecules, comparable with the experimental data [117]. B3LYP, Hartree-Fock HF and other method have been tested, in a comparative study on stability of fullerenes [118]. Magnetic susceptibility is also useful in this respect; values of exaltation of magnetic susceptibility for the basic polycyclic aromatic hydrocarbons (PAHs) have been collected in ref. [119]. Magnetic susceptibility can be calculated e.g., by B3LYP method, that properly indicate the trend of stabilization in PAHs [120].

## 7. Conclusions

In this review article, we presented computational arguments in supporting the possible existence of some not yet synthesized molecules, involved in nanosystems. Modeling small molecules enables one to build novel assemblies that may explain observed aggregates, this being not a trivial task of theorists. Four ideas have been detailed:

Aromaticity of new fullerenes, patched with flowers of 6-and 8-membered rings, was discussed in terms of HOMA and NICS criteria. The spanned fullerenes patched by coronene and sumanene motifs evidenced clear electronic differences inside to outside the cage, as resulted from the NICS calculations. The calculated aromaticity parameters: HOMA, NICS, magnetic susceptibility, formation enthalpy, electronegativity, total hardness, electrophilicity and reactivity Fukui functions, computed on Hartree-Fock and DFT optimized molecular structures, provided a complex image on the electron distribution and stability of these yet hypothetical fullerenes, in agreement with the experimental data for the consisting patches, collected in the literature (as real molecules).

Polybenzene networks have been presented, from construction to energetic and vibrational spectra computations. The energetics and spectra of some repeating units, monomers, dimers, oligomers, involved in the construction of 3-periodic or 1-periodic polybenzene nanostructures, have been presented with the aim of helping experimentalists in eventual syntheses. The aromaticity of benzene patches (*i.e*., hexagonal rings) in polybenzenes seems to be rather close to that of the isolated benzene molecule, as suggested by their small distortions to the planarity.

P-type crystal networks have been designed in several decorations; the reviewed data presented some zeolite-like 3-periodic nanostructures constructed with sumanene or derived patches, for which energetics and vibrational specta have been computed; data supported the idea that such “ordered schwarzites” could be real molecular crystals.

Construction and stability evaluation (at DFTB level of theory) of some exotic allotropes of diamond D_5_, involved in hyper-graphenes, was presented at the end of this review. Substructures of the hyper-diamond D_5_ was shown to form 3-periodic networks, as in D_5_-anti allotropes, or 1-periodic quasicrystal allotropes, as in D_5_-syn structures. Also, the D_5_-syn-allotrope can form hyper-graphenes, e.g., by cutting with an electron beam. In view of understanding the reliability of various computational approaches, comparative study was done using Hartree-Fock, Density Functionals and the semi-empirical DFTB method. Conclusion was that, in spite of some differences, the three approaches are useful in predicting the stability of substructures involved in nanosystems, DFTB being important in ordering some large atom number structures. Even the majority of the presented structures are yet hypothetical ones, they represent slides of a scientific dream.
